# Screening for Staphylococcal Superantigen Genes Shows No Correlation with the Presence or the Severity of Chronic Rhinosinusitis and Nasal Polyposis

**DOI:** 10.1371/journal.pone.0009525

**Published:** 2010-03-05

**Authors:** Frédéric Heymans, Adrien Fischer, Nicholas W. Stow, Myriam Girard, Zacharias Vourexakis, Antoine Des Courtis, Gesuele Renzi, Elzbieta Huggler, Stefan Vlaminck, Pierre Bonfils, Ranko Mladina, Valerie Lund, Jacques Schrenzel, Patrice François, Jean Silvain Lacroix

**Affiliations:** 1 Rhinology-Olfactology Unit, Department of Otorhinolaryngology and ENT Surgery, Geneva University Hospital, Geneva, Switzerland; 2 Genomic Research Laboratory, Infectious Diseases Unit, Department of Internal Medicine, Geneva University Hospital, Geneva, Switzerland; 3 Central Laboratory of Bacteriology, Department of Medical Genetics and Laboratory Medicine, Geneva University Hospital, Geneva, Switzerland; 4 AZ Sint-Jan AV, Bruges, Belgium; 5 Department of ENT Head and Neck Surgery, Georges Pompidou European Hospital, Paris, France; 6 Department of ENT Head and Neck Surgery, Clinical Hospital Center, Zagreb, Croatia; 7 UCL EAR Institute of Laryngology and Otology, London, United Kingdom; Hospital of the University of Pennsylvania, United States of America

## Abstract

**Background:**

Staphylococcus aureus secretes numerous exotoxins which may exhibit superantigenic properties. Whereas the virulence of several of them is well documented, their exact biological effects are not fully understood. Exotoxins may influence the immune and inflammatory state of various organs, including the sinonasal mucosa: their possible involvement in chronic rhinosinusitis has been suggested and is one of the main trends in current research. The aim of this study was to investigate whether the presence of any of the 22 currently known staphylococcal exotoxin genes could be correlated with chronic rhinosinusitis.

**Methodology/Principal Findings:**

We conducted a prospective, multi-centred European study, analysing 93 Staphylococcus aureus positive swabs taken from the middle meatus of patients suffering from chronic rhinosinusitis, with or without nasal polyposis, and controls. Strains were systematically tested for the presence of the 22 currently known exotoxin genes and genotyped according to their *agr* groups. No direct correlation was observed between chronic rhinosinusitis, with or without nasal polyposis, and either *agr* groups or the presence of the most studied exotoxins genes (*egc*, *sea*, *seb*, *pvl*, *exfoliatins* or *tsst-1*). However, genes for enterotoxins P and Q were frequently observed in nasal polyposis for the first time, but absent in the control group. The number of exotoxin genes detected was not statistically different among the 3 patient groups.

**Conclusions/Significance:**

Unlike many previous studies have been suggesting, we did not find any evident correlation between staphylococcal exotoxin genes and the presence or severity of chronic rhinosinusitis with or without nasal polyposis.

## Introduction

Chronic rhinosinusitis (CRS) is an inflammation of the upper airway mucosa and is often associated with asthma. The prevalence of CRS is about 16% of the general population in western countries [1]. It is associated with a significant reduction of patients' quality of life and high economic costs [1].

A comprehensive definition of CRS was described in the recent EPOS article [1]: CRS is an inflammation of the nose and paranasal sinus mucosa, characterised by persistent symptoms for over 12 weeks. There must also be signs on nasal endoscopy and/ or CT-scan. The development of nasal polyposis (CRSwNP) can be an additional clinical finding in approximately 20% of CRS cases and is considered to be the ultimate development of the disease [1], [2].

The pathophysiology of CRS is not fully understood and is most likely multifactorial. Systemic host factors, including genetic background [3], [4], host immunological profile and environmental factors are thought to predispose to the development of CRS [2]. According to several recent studies, bacterial colonization and, especially bacterial products such as exotoxins from Staphylococcus aureus (SA), could be implicated in the pathogenesis of CRS with or without NP [5]–[8].

Staphylococcus aureus produces a plethora of toxins associated with several lethal human infections [9]–[11]. Exotoxins from SA are exported to the extracellular space, where they can act as superantigens. Some of these exotoxins have been identified as important contributors in food intoxications [9]–[12] and were thus called enterotoxins. Others have been found to be potential markers of severe infections [13], [14]. They play a role not only in classical staphylococcal infections, but also during non-infectious diseases [15]. Most SA exotoxins (SE) are pore-forming molecules: the exfoliatins are responsible for the necrotising skin rash in the newborn [9]; the toxic-shock staphylococcal toxin (TSST-1) is known to induce shock in the menstrual toxic-shock syndrome [9]–[11]; the Panton-Valentine leukocidin (PVL) is responsible for necrotizing haemorrhagic pneumonia, skin and osteoarticular infectious manifestations [16]–[18]. However, 80% of healthy subjects show the presence of anti-TSST-1 antibodies in their plasma; in contrast, similar antibodies are found in only 20% of patients showing toxin-related septic shock [19].

Staphylococcal exotoxins can act as superantigens, which are able to directly stimulate T cells, thus producing an intense polyclonal immune response. SE can bind to the side of T cell MHC-II V beta domains receptors [20], inducing a potent immune response without the need of antigen-specific recognition. Recent studies suggest that certain SE (enterotoxins A, B, C, D and TSST-1) may act as superantigens in the pathogenesis of CRSwNP, worsening inflammation by inducing an immunological response [5]–[8], [21]. A normal humoral immune response might also participate by adding antigen-antibody stimulated inflammation [5]. The severity of CRSwNP could be associated with an immunological response toward exotoxins released by SA colonizing the nose [5]–[8], [21]. This hypothesis was supported by the absence of exotoxins in non-polypoid mucosa from 11 patients, screened for staphylococcal enterotoxins A, B, C, D & TSST-1 [8].

A possible involvement of different SE in nasal mucosal inflammation has been proposed. Our study was designed to investigate whether a correlation between the presence of SA exotoxin genes (*se*) and CRS could be observed. Hence, we designed a prospective, multi-centred European study, to minimize bias due to local SA epidemiology. As previous reports have shown the frequent association of CRSwNP, asthma and non-steroid anti-inflammatory drugs (NSAID) intolerance (known as Widal-Lermoyez syndrome [22] or Samter's Triad [23]), we also collected data on the presence of these conditions in all patients.

## Methods

Inclusion criteria were a positive middle meatus swab for SA in patients undergoing nasal endoscopic surgery during the study period. Exclusion criteria were based on a positive medical history of allergy, current upper respiratory tract infection and systemic corticotherapy during the last 6 months. All CRS patients, with or without polyps, were under topical corticotherapy for more than six months.

Among 268 patients, 214 were recruited because they were suffering from CRS, with or without NP; 54 patients were recruited as controls, according to the criteria listed below. 93 patients out of the 268 were positive for intranasal SA carriage and were therefore included in the study: 71 suffering from CRS and 22 controls. Ages ranged between 16 and 82 years, encompassing 39 females and 54 males, recruited in Paris (France) (n = 27), Bruges (Belgium) (n = 9), London (UK) (n = 2), Zagreb (Croatia) (n = 6) and Geneva (Switzerland) (n = 49: 27 CRS and 22 SA healthy carriers as controls).

The patients were divided into three groups. The first group was composed of 55 patients suffering from CRSwNP, the second group contained 16 patients suffering from CRS without NP (CRSsNP) and the third group consisted of 22 controls, from. All patients in the first two groups were diagnosed with CRS according to the EPOS definition [1]. All patients in the control group underwent nasal endoscopic surgery for non-CRS related conditions. Nasal polyposis severity was evaluated by endoscopic examination, following the Malm classification (stage 1 = polyps present in the middle meatus; stage 2 = polyps extending below the middle turbinate; stage 3 = polyps reaching the floor of the nasal cavity) [24]. A history of asthma (categorised into moderate or severe) and Widal syndrome was noted.

Bacteriological samples were obtained from all patients by swabbing the middle meatus mucosa under endoscopic control. A Gram stain was performed and the quantity of SA bacteria (QB) was categorised into 3 groups: “low” was defined by the presence of colonies only in the first streaking quadrant on a Petri dish, “moderate” when colonies were present in the first two streaking quadrants and “abundant” whenever colonies were found in the third streaking quadrant.

Identification of SA was performed according to CLSI recommendations [25]. Each sample was tested for the presence of the *femA* gene, a SA-specific gene in order to confirm species identification and to assess the quality of further PCR amplifications. PCR was also used to detect the presence of the 22 currently known SA exotoxins genes and 2 pseudogenes. These determinations included PVL, TSST-1 and exfoliatins A and B genes, as well as 18 enterotoxin genes (*se*). The 2 non-functional pseudogenes, *yent1* and *yent2*, can rearrange together to yield the functional enterotoxin gene *seu* [26]. Their presence was not considered in the final analysis; rather, we only considered the presence of the functional gene. Because they usually co-transfer between bacteria, *seg, sei, sem, sen, seo seu* form a cluster named *egc* (enterotoxin gene cluster). Likewise, *sed* and *sej* usually co-transfer and, so, form another cluster.

We analysed the data to investigate a possible correlation between the presence and severity of CRS and the presence and quantity of both bacteria and *se*.

### DNA Preparation

Genomic DNA was extracted using Dneasy kit (QIAGEN, Hilden, Germany) from isolated colonies freshly grown on Mueller Hinton agar plates (Difco). Each strain was suspended in 500 µl Tris-EDTA buffer (TE, 10mM Tris and 1 mM EDTA), then washed twice by centrifugation for 10 minutes at 4000 g. The pellet was suspended in 200 µl TE supplemented by 100 µg of lysostaphin (Ambicin, Applied Microbiology, Tarrytown, NY), and incubated for 10 minutes at 37°C. DNeasy spin columns were then used following manufacturer's recommendations. DNA concentration and purity were assessed by using NanoDrop ND-1000 spectrophotometer (NanoDrop Technologies, Inc., Rockland, DE). For clinical isolates tests, fast DNA extraction was performed using glass beads (in Tris-EDTA buffer) and vortex agitation [26]. 5 µl of crude lysate were used to performed PCR amplifications. Control experiments showed that rapid extraction yielded similar results to purified DNA samples [26].

### Primers and PCR Amplification

SA genotyping was performed according to accessory gene regulator (*agr*) groups, using a previously published real-time multiplex PCR assay [27]. This consists of a quadruplex PCR amplification allowing discrimination of the 4 different alleles which define the *agr* groups of SA [28]. Enterotoxins were identified by using 4 PCR multiplex amplifications [29]. 1µl of each PCR mixture was loaded in a BioAnalyzer 2100 device using DNA 1000 chip, yielding to an outstanding resolution from 25-1000 bp. Results were analyzed using specific software previously described in the study of SA by VNTR [30]. Briefly, BioAnalyzer output files containing raw fluorescence data were exported and processed to discriminate peaks from background signals. Exfoliatin A and B genes (*etA* and *etB*), *tsst* and *pvl* were identified as formerly described [27].

**Rapid genotyping** was performed using a recently published multilocus variable number of tandem repeats assay (MLVA) using 10 primer pairs [30], [31] and a microcapillary electrophoresis system for the rapid evaluation of the MLVA profile. MLVA genotyping is a rapid methodology allowing the identification of the bacterial genetic background and the relatedness between isolates with a discrimination power at least comparable to that of PFGE [30].

### Statistical Analysis

The “Fisher Exact Test” was used to calculate the P-values reflecting the difference between groups containing categorical data. The “Mann Whitney U Test” was used for the same purpose with quantitative data and a chi-squared test for 3 comparison groups. A p-value inferior to 0.05 was considered as significant.

### Ethic Statement

This study was approved by the Geneva University Hospital Ethics Committee. Informed consent was obtained: written in Geneva and Paris and verbal in London, Bruges and Zagreb. All patients were informed that their swab results and clinical findings would be kept anonymous. The data transmitted to the last author concerned the state of CRS and grade of polyposis, the bacterial load, the grade of asthma and any non steroid anti-inflammatory drugs intolerance.

## Results

The presence of SA was documented by culture in 71/214 (33.2%) patients suffering from CRS, among whom 55 CRSwNP and 16 CRSsNP, and in 22/54 controls (40.7%): these rates are similar to the SA intranasal carriage in the general population [13] as previously described by our group [32], [33]. The proportions of SA intranasal carriers in the CRS group were of 44.3% in Paris (n = 27/61 CRS), 32.1% in Bruges (n = 9/28 CRS), 50% in London (n = 2/4 CRS), 18.2% in Zagreb (n = 6/33 CRS) and 30.6% in Geneva (n = 27/88 CRS). 175/214 patients were negative for SA intranasal carriage, among whom 143 CRS patients, 91 CRSwNP and 52 CRSsNP, and 32 controls.

Among the 214 CRS patients with and without polyps, 71 were intranasal *Staphylococcus aureus* carriers and 143 were non-carriers; in the control group, 22 were intranasal SA carriers and 32 non-carriers: no significant difference was found between those groups (p-value = 0.188 - Fisher Exact Test). We also analyzed the subgroups separately, following their clinical status (CRSwNP, CRSsNP) and controls. Within the CRSwNP patients group, 55 were intranasal SA carriers and 91 non-carriers; within the CRSsNP patients group, 16 were intranasal SA carriers and 52 non-carriers. There was no significant difference between those 3 groups either (p-value = 0.075 - Chi-Squared Test).

**Figure 1:**
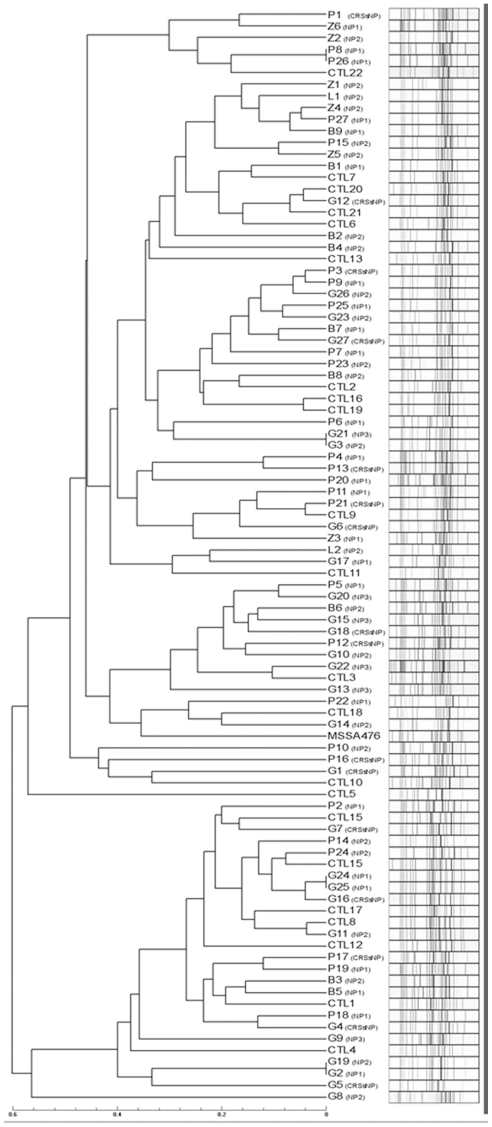
Genotyping tree. MLVA genotyping of isolates recovered from patients suffering chronic rhinosinusitis from five different geographic locations (B. Brugg; Z. Zagreb; P. Paris; L. London; G. Geneva). Control isolates (CTL) from Geneva patients are also depicted (a single isolate per patient is shown). Categories are indicated between brackets. Scale displays the relations between isolates. Isolates with a maximal distance 0.1 [^30^] are considered as clonal. CRSsNP = Chronic Rhinosinusitis without Nasal Polyposis group; NP1-2-3 = Nasal Polyposis grade 1-2-3 groups; Ctl = control group.

Regarding the SA intranasal carriers, accessory gene regulator (*agr*) typing showed a similar distribution of each of the 4 alleles in all patients studied. The most abundant *agr*-type identified overall was type I, irrespective of the geographical origin of the SA strains. Rapid genotyping failed to identify clusters matching the geographical origin of the isolates (cf. figure 1). Thus, considering the three populations subjected to MLVA analysis, we observed a large diversity of unrelated isolates, derived from various genetic backgrounds, with the largest clusters containing a maximum of 3 strains, without correlation with any group of patients.

**Figure 2:**
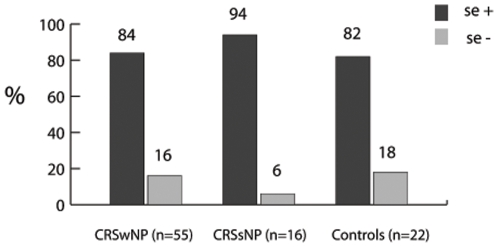
Rates of patients carrying S. aureus. Percentage, per group of patients, of *S. aureus* with (se +) or without (se −) exotoxin gene.

Each of the 3 patient groups contained SA strains with and without *se*. The distribution across the patient groups is illustrated in figure 2. The quantity of each *se* found in each group and subgroup is displayed in Table 1. At least one *se* was detected in 79/93 patients (84.9%), including 46/55 with CRSwNP (83.6%), 15/16 with CRSsNP (93.7%) and 18/22 controls (81.2%) (Table1). There was no statistically significant difference between these rates in CRSwNP patients and controls (p = 0.253), CRSwNP and CRSsNP patients (p = 0.22) or the CRSsNP patients and controls (p = 0.233). A more detailed analysis of the CRSwNP group was performed by dividing it into 3 subgroups, according to the stage of nasal polyposis (stage 1 = NP1, stage 2 = NP2, stage 3 = NP3). At least one *se* was detected in 24/25 patients with NP1 (96%), 20/24 with NP2 (83%) and only 2/6 with NP3 (33%). There was no statistically significant difference in rates between groups NP1 and NP2 (p = 0.15). NP3 was too small to be analyzed.

**Table 1:** Staphylococcus aureus genome screening.

												exotoxin genes																
									enterotoxin genes																	*agr*		
				*egc*				*dj cluster*				other enterotoxin genes																
	Patients per group	*seg*	*sei*	*sem*	*sen*	*seo*	*seu*	*sed*	*sej*	*sep*	*seq*	*sea*	*seb*	*sec*	*see*	*seh*	*sek*	*sel*	*ser*	*tsst-1*	*pvl*	*etA*	*etB*	*Total*	*I*	*II*	*III*	*IV*
**CRSwNP**	**55**	42	42	42	39	42	42	1	1	9	8	10	1	9	0	6	0	10	2	10	1	1	0	318	26	18	11	0
*** NP1***	***25***	*21*	*21*	*21*	*21*	*21*	*21*	*0*	*0*	*7*	*5*	*5*	*1*	*3*	*0*	*4*	*0*	*4*	*0*	*4*	*1*	*1*	*0*	*161*	*9*	*11*	*5*	*0*
*** NP2***	***24***	*19*	*19*	*19*	*17*	*19*	*19*	*1*	*1*	*2*	*3*	*5*	*0*	*6*	*0*	*1*	*0*	*6*	*2*	*5*	*0*	*0*	*0*	*144*	*14*	*5*	*5*	*0*
*** NP3***	***6***	*2*	*2*	*2*	*1*	*2*	*2*	*0*	*0*	*0*	*0*	*0*	*0*	*0*	*0*	*1*	*0*	*0*	*0*	*1*	*0*	*0*	*0*	*13*	*3*	*2*	*1*	*0*
**CRSsNP**	**16**	13	14	14	13	13	13	0	0	1	1	1	0	3	0	2	0	1	0	2	0	0	0	91	8	3	4	1
** Ctl**	**22**	18	18	18	18	18	18	0	0	0	0	3	0	2	0	2	0	3	0	7	0	0	0	125	9	6	7	0
** Total**	**93**	73	74	74	70	73	73	1	1	10	9	14	1	14	0	10	0	14	2	19	1	1	0	534	43	27	22	1
**p-value CRSwNP/Ctl**		0.22	0.22	0.22	0.15	0.22	0.22	0.71	0.71	**0.039**	**0.033**	0.46	0.71	0.33	1	0.59	1	0.46	0.51	0.16	0.71	0.71	1	0.53				

Number of patients carrying the different staphylococcal exotoxins and their agr-related grouping; the p-values for each se were calculated using the Fisher Exact Test; the p-value for the comparison of the total number of se between the groups was calculated using the Mann Whitney U Test. (CRSwNP = Chronic Rhinosinusitis with Nasal Polyposis group; CRSsNP = Chronic Rhinosinusitis without Nasal Polyposis group; NP1-2-3 = Nasal Polyposis grade 1-2-3; Ctl = control group; agr = accessory gene regulator (genotyping marker); se = staphylococcal enterotoxin gene; etA and etB = exfoliatins A and B genes; tsst-1 = Toxic Shock Staphylococcal Toxin-1 gene; pvl = Panton-Valentine leukocidin gene).

**Figure 3:**
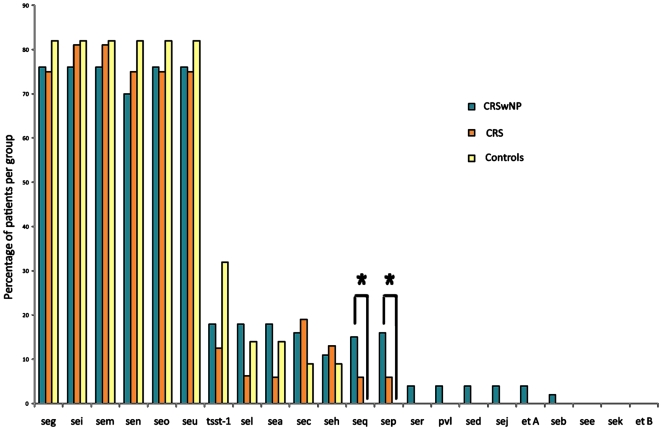
Identified exotoxins. Number of different staphylococcal exotoxins identified in the CRSwNP, CRSsNP and control groups, in percentage per groups' population, in order to compare the three groups equally.

There was no geographical difference in the carriage of any particular *se*. The different types of *se* were equally distributed between women and men. Figure 3 shows the percentages of each gene detected in the three groups. No individual *se* was always present in all of the 79 patients in which *se* were detected. The most frequently detected *se* were those of the *egc* (enterotoxin gene cluster: *seg, sem, sen, sei, seo, seu*), in 75/79 patients (94.9%). All patients in the CRS and control groups who had *se* detected had the *egc* present, while the only four patients without genes of this cluster (but with other *se* present) were in the CRSwNP group. Other commonly detected *se* were *tsst-1* (19/79; 24%), *sea, sec, sel* (14/79; 17.7%), *sep*, *seh* (10/79; 12.7%) and *seq* (9/79; 11.4%). *see*, *sek* and *etB* were not detected in any patient. All other exotoxins were detected in only 1 or 2 patients (Table 1).

The detection rates of each *se* in the CRSwNP and control groups were compared by the Fisher exact test. Statistically significant differences were found for *sep* (p = 0.039) and *seq* (p = 0.033), both of which were present in 8 and 9 CRSwNP patients, respectively, but not found in any control patient. There was no difference in the detection rates of any other *se* between these groups (Table 1).

Overall, there were 318 *se* detected in the 55 patients with CRSwNP, 85 detected in the 16 patients with CRSsNP and 125 detected in the 22 controls. Using the Mann Whitney U Test, there was no statistically significant difference between the overall numbers of *se* detected in the CRSwNP and control groups (p = 0.532), the CRSwNP and CRSsNP groups (p = 0.259) and the CRSsNP and control groups (p = 0.492) (Table 2). The median number of *se* per patient was 7 in the CRSwNP group (range 0–11), 6.5 in the CRS group (range 0–8) and 6 in the controls (range 0–8). Hence, no SA strain carried all of the 22 known *se*.

**Table 2:** Correlations between groups.

p-value CRSwNP/CRS	0.259
p-value CRSwNP/Ctl	0.532
p-value CRS/Ctl	0.492
p-value NP3/NP2	**0.036**
p-value NP3/NP1	**0.009**
p-value NP2/NP1	0.275

Calculations made considering the total amount of exotoxin genes detected in each group (statistical analysis: Mann Whitney U Test): the p-value is relevant for NP1 and NP2, against NP3.

The CRSwNP group revealed 161 *se* detected in the 25 NP1 patients, 144 in the 24 NP2 patients and 13 in the 6 NP3 patients, with no significant difference between the overall number of *se* detected in the NP1 and NP2 (p = 0.275) (Table 2). The NP3 group consisted of 6 patients, only 2 of which had detectable *se*, suggesting that some patients can develop the most severe stage of the disease in the absence of a single exotoxin gene.

There were significantly more bacteria found in CRSwNP patients when compared to both controls (p<0.0001) and CRSsNP (p = 0.016) patients. CRS patients had significantly more bacteria than controls (p = 0.013). There was no statistically significant difference in the quantity of bacteria when comparing NP1 with NP2 patients (p = 0.258).

## Discussion

Five main observations were obtained from this study. Firstly, we did not find significant differences between CRSwNP, CRSsNP and control groups regarding the number of patients carrying intranasal SA or not.

Secondly, we did not find significant differences between CRSwNP, CRSsNP and control groups regarding the number of patients who had at least one *se* detected. Thirdly, there was no difference between these groups in the overall quantities of *se* detected. Fourthly, *sep* and *seq* were detected in 16% (9/55) (p = 0.039) and 14.5% (8/55) (p = 0.033) respectively of CRSwNP patients, but never in controls. It is, however, difficult to draw conclusions from this finding, since these two *se* were only present in a small minority of symptomatic patients and no study is yet available regarding their biological properties in airways. Finally, the frequency of all other individual *se* was similar between groups, suggesting that the correlation between the presence of any particular *se* and the severity of CRS remains unclear. In other words, no single exotoxin gene or cluster was found to be associated with CRS, with or without NP.

When analysing individual *se*, the *egc* constituted the most frequently identified group of genes encoding for SE, which is in accordance with a recent study [33]. Two isolates were carrying an incomplete *egc*, lacking the *seg*, an event which has been described previously [15]. The *egc* was equally distributed across all SA carriers groups: thus, no correlation could be found between the presence of the *egc*-gene cluster and clinical findings. This was also the case for *sea, seb, sec, sed and tsst-1.* There were 9 CRSwNP patients in whom no *se* was detected. These findings suggest that *se* are not essential to the development of CRSwNP and actually, are not even more common in CRSwNP than in controls that carry SA. This may contradict previous studies which claimed that SE were likely to be involved in the pathogenesis of CRSwNP [2], [5], [6], [8], [21]. As previously reported, SE may change the balance of inflammatory mediators to worsen inflammation at a histological level [18], [34], but our data suggest there is no evidence to link this to a clinical change. Along similar lines, there was no significant difference in the overall quantity of *se* detected in NP1 and NP2 patients groups. Assessment of the role of *se* in the NP3 group remains limited by its small number of patients. It should be noted that out of the 9 CRSwNP patients where no *se* was detected, 1 patient was in NP1, 4 were in NP2 and 4 were NP3 subgroups.

We tested for the largest number of individual exotoxins and our *se* prevalence rate was the highest, at 85%. Some studies have found that exotoxins changed the balance of inflammatory mediators to worsen inflammation [18], [34]. While this may be indeed the case, our data suggests that the degree of histological changes is not associated with NP1 and NP2, following endoscopic observations. A significant correlation was found linking the presence of *sep* and *seq* exclusively to the CRSwNP group:. Since these two *se* have not yet been described in recent studies on CRS, they would deserve further investigations. In the present study, 7/55 CRSwNP patients had SA carrying *sep* and *seq* simultaneously. This probably reflected two distinct genetic transfers in a single bacterium, although they are not known to form a cluster. While recent studies suggest a role for *sep* [34] and *seq* [35] in food poisoning, there is no publication yet on their potential role in CRS. Two isolates were carrying an incomplete *egc*, lacking the *seg*, as it has been already described [15].

There was no significant difference in the quantity of all *se* detected in NP1 and NP2 patients groups. Assessment of the role of *se* in the NP3 group remains however limited by the small number of patients included in this group. It should be noted that out of the 9 CRSwNP patients where no *se* could be detected, 1, 4 and 4 were observed in NP1, NP2 and NP3, respectively. Altogether, these observations suggest that the role of *se* in the pathogenesis and severity of CRS remains to be clarified.

Among the 3 patients with Widal syndrome in the NP3 group, 2 had no detectable *se*, suggesting that this pathological entity is not strictly dependent on the presence of *se*. This syndrome is associated with a defined genetic background; the development of CRS with or without NP in general could also include different genetic susceptibility to the very same SA and their exotoxins, as well as to other unknown pathological pathways.

Recent evidence has suggested that inflammatory processes in CRS could be related to the host immune response to bacterial components present in the nasal mucosa [3], [33]. The presence of activated T lymphocytes, within the nasal mucosa of patients with CRSwNP, and IgE directed against SE has been suggested as evidence for a role for exotoxins in the pathogenesis of this disease. Intranasal bacteria, with or without exotoxins, could have various effects in different individuals, depending on genetics and other factors within the host response.

To our knowledge, the present study is the largest of its type to report on the correlation of SA exotoxin genes in CRS with and without NP. Compared to other similar studies, it tested the largest number of patients for the greatest number of individual *se*. This may have contributed to the fact that our *se* prevalence rate in SA carriers was the highest reported, at 85%. Interestingly, only 71 of 214 patients suffering from CRS were carrying SA and only 61 had *se* present in the SA strains. It is reasonable to expect that fewer still would go on to have *se* translated into exotoxins proteins and secreted into the extracellular compartment, where they could become pathogenic.

Recent findings also suggest that CRS could be related to the host genetic background and immune response to bacterial components present in the nasal mucosa [3], [33]. Intranasal bacteria, with or without exotoxins could have various effects in different individuals, depending on the host response.

MLVA analysis suggested that neither the geographical origin nor the genetic background of SA isolates was associated with a specific population. A greater number of bacteria was found in CRSwNP when compared to CRS, as well as in CRS when compared to controls. There was no statistical difference in the number of bacteria present in NP1 and NP2 patients. The presence of SA and, hence, its exotoxins in the diseased population might be circumstantial. Indeed, mucosal inflammation of any origin is known to be associated with mucociliary dysfunction, which may lead to bacterial colonisation due to decreased bacterial clearance [36]–[38].

The present study detected SA genes that encode exotoxins, but not the exotoxins themselves. It is well known that the sensitivity issue is of paramount importance for providing reliable detection of enterotoxins at the protein level and that currently available techniques are neither sensitive enough, nor can they detect all known *S. aureus* exotoxins. Furthermore, it would be difficult to correlate *in vitro* testing with *in vivo* conditions, which most likely involve multiple environmental factors that affect enterotoxin gene regulation. By identifying exotoxin genes, we aimed at exploring which individual exotoxins were present in the different patient groups as well as determining their frequency, making the assumption that a preserved gene is most likely expressed. Under specific conditions allowing gene expression, the amount of secreted exotoxin is dependent on the number of bacteria present in tissue. According to previously published studies, the presence of SA may be underestimated due to the intracellular location of bacteria [39], the presence of biofilm or that of mucus [40], [41]. In this study, we used conventional techniques to grow SA and such methods may not be optimally sensitive to detect the presence of specific bacterial modes of growth, like intracellular growth or biofilms. Thus, culture positivity may vary over time due to such technical challenges [39]. In this study, there was no statistical difference in the number of bacteria present in NP1 and NP2 patients. A greater quantity of bacteria may be a consequence, rather than a cause of the inflammation, or may be both. Thus, the possible contribution of SA exotoxins in the pathogenesis of CRS remains to be clarified since only 71/214 patients suffering from this disease were carrying SA.

To date, studies suggesting a role of SA exotoxins in CRS, as well as in other diseases, are based on gene detection. As recently illustrated by Katsuhiko Omoe *et al.*, « most of the *S. aureus* isolates harboring *seg* and about 60% of the isolates harboring *sei* did not produce a detectable level of SEG or SEI » [42]. This was also recently illustrated by Young-Duck and colleagues [43] who reported a good correlation between gene presence and gene expression, but a poor correlation between gene expression or presence and protein presence. The possible role of superantigens in the development of CRS is supported by the presence of activated Tvb lymphocytes as well as IgE in the nasal mucosa of patients with NP. However, their role in the constitution of NP has not yet been clarified. Taken together, this suggests that we may overestimate the prevalence of secreted exotoxins as well as that of their pathogenic role.

In summary, we did not find any correlation between the presence of Staphylococcus aureus and the development of CRS, with or without polyps. Our study focused on exotoxin genes of SA carriers: we did neither find any correlation between the presence of any particular *se*, nor large numbers of *se,* and CRS, with or without NP. In addition, there was no evidence that *se* may influence the severity of the disease and development of nasal polyposis. Nevertheless, the genes for recently discovered enterotoxins P and Q were detected for the first time in CRS and CRSwNP: further research may provide new insights into their role. It has been suggested that theoretically, an infinite number of SE may arise from complex immune processes which stimulate genetic mutations in SA [11]. New exotoxins may remain to be discovered.
